# Legacy and Emerging Per- and Polyfluoroalkyl Substances: Analytical Techniques, Environmental Fate, and Health Effects

**DOI:** 10.3390/ijms22030995

**Published:** 2021-01-20

**Authors:** Richard A. Brase, Elizabeth J. Mullin, David C. Spink

**Affiliations:** 1 Laboratory of Organic Analytical Chemistry, Wadsworth Center, New York State Department of Health, Albany, NY 12237, USA; rbrase@albany.edu (R.A.B.); elizabeth.mullin@health.ny.gov (E.J.M.); 2 Department of Environmental Health Sciences, School of Public Health, University at Albany, State University of New York, Rensselaer, NY 12144, USA

**Keywords:** per- and polyfluoroalkyl substances, PFAS, HFPO-DA, GenX, mass spectrometry, landfills, biomonitoring, biotransformation, immunotoxicity, carcinogenicity

## Abstract

Due to their unique chemical properties, per- and polyfluoroalkyl substances (PFAS) have been used extensively as industrial surfactants and processing aids. While several types of PFAS have been voluntarily phased out by their manufacturers, these chemicals continue to be of ecological and public health concern due to their persistence in the environment and their presence in living organisms. Moreover, while the compounds referred to as “legacy” PFAS remain in the environment, alternative compounds have emerged as replacements for their legacy predecessors and are now detected in numerous matrices. In this review, we discuss the historical uses of PFAS, recent advances in analytical techniques for analysis of these compounds, and the fate of PFAS in the environment. In addition, we evaluate current biomonitoring studies of human exposure to legacy and emerging PFAS and examine the associations of PFAS exposure with human health impacts, including cancer- and non-cancer-related outcomes. Special focus is given to short-chain perfluoroalkyl acids (PFAAs) and ether-substituted, polyfluoroalkyl alternatives including hexafluoropropylene oxide dimer acid (HFPO-DA; tradename GenX), 4,8-dioxa-3H-perfluorononanoic acid (DONA), and 6:2 chlorinated polyfluoroethersulfonic acid (6:2 Cl-PFESA; tradename F-53B).

## 1. Introduction to Per- and Polyfluoroalkyl Substances (PFAS)

Per- and polyfluoroalkyl substances (PFAS) constitute a broad class of man-made chemicals that contain at least one perfluoroalkyl moiety in which all H substituents on a C atom have been replaced by F [1]. The creation of these non-polar, perfluoroalkyl chains is accomplished by two primary manufacturing processes: electrochemical fluorination (ECF) and telomerization [1]. In addition to a perfluoroalkyl chain, most PFAS contain a polar functionality such as a carboxylic or sulfonic acid group in the respective cases of perfluorocarboxylic acids (PFCAs) and perfluorosulfonic acids (PFSAs). The unusual strength of the C–F bonds in PFAS make them extremely resistant to degradation, while their hydrophobic and lipophobic nature gives them unique, surface-active properties. The chemical stability and amphiphilic nature of these compounds gave rise to their use in several industrial and consumer-product applications that have emerged since the late 1940s [2]. For example, PFAS have been used in creating oil- and grease-resistant food packaging, non-stick cookware, water- and stain-resistant textiles, aqueous fire-fighting foams (AFFFs), and numerous other products. It was estimated in 2015 that at least 3000 types of PFAS have been on the global market for use in these types of applications [3].

**Figure 1:**
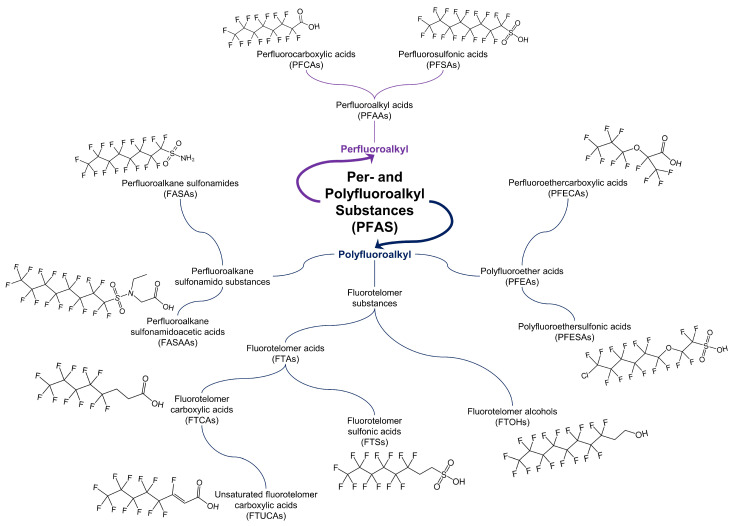
Examples of various PFAS subclasses (with non-polymeric examples shown) and the chemical structures of compounds representative of these subclasses.

Due to their widespread use and chemical stability, PFAS are highly persistent in the environment and can be ubiquitously detected in numerous matrices including water, soil, plants, animals, foodstuffs, and human serum [4,5,6]. The environmental persistence of these compounds in combination with the ever-growing body of evidence of their potential negative health effects led to regulatory efforts and voluntary initiatives calling for decreased use of certain long-chain perfluoroalkyl acids (PFAAs) (fluorinated carbons ≥7 for PFCAs; ≥6 for PFSAs [7]) starting in 2000 [8,9]. This included the voluntary phase out of perfluorooctanesulfonic acid (PFOS), perfluorooctanoic acid (PFOA), and their precursors during the period 2000–2002 by the leading global manufacturer at the time [8,9]. However, owing to the usefulness of these compounds in numerous industrial processes, manufacturers began to replace long-chain PFAAs with alternative PFAS, including short-chain PFAAs (fluorinated carbons ≤6 for PFCAs; ≤5 for PFSAs) and newer, polyfluorinated alternatives [10,11]. For the purpose of this review, the term “legacy” refers to long-chain PFAAs that have been phased out of production in numerous developed nations, while the terms “emerging” and/or “alternative” refer to short-chain PFAAs and polyfluorinated compounds used as replacements for legacy PFAS. Figure 1 depicts some of the PFAS subclasses (non-polymeric) and the chemical structures of specific compounds representative of those subclasses.

With the introduction of newer, emerging PFAS and the persistence of legacy compounds in the environment, PFAS continue to be a topic of great interest and ongoing research in the scientific community. The objective of this review is to provide an overview of the current status of PFAS research in the realms of analytical chemistry, environmental science, epidemiology, and toxicology. In this review, we summarize routine and state-of-the-art techniques for PFAS analysis, including both targeted and untargeted methods of mass spectrometry. Additionally, we describe the fate of PFAS in the environment, including sources of contamination, distribution throughout various environmental matrices, and potential for transformation in those matrices. Moreover, we review the status of human health-related PFAS research with a focus on biomonitoring, metabolic fate, toxicology, and cancer epidemiology. To prepare this review, PubMed and other available databases were queried using appropriate search terms and combinations. The purpose of this review is to highlight the current knowledge and advances in the study of PFAS, but it is not to serve as a compilation of data for further analysis. Finally, we provide suggestions for the future directions of PFAS research based on insights from the literature outlined in this review. The names, acronyms, and Chemical Abstracts Service Registry Number (CASRN) of specific PFAS discussed in this work can be found in Table S1 of the Supplementary Material.

## 2. Analytical Techniques for PFAS

The ability to measure PFAS in various matrices serves as the foundational basis for studying the behavior of these compounds in the environment and their effects on human health. In addition, as regulatory bodies set guidelines on maximum permissible levels of PFAS, the accurate detection of these compounds becomes increasingly important. For these reasons, analytical methods are constantly improving to reach lower detection limits, measure a greater number of analytes, and discover uncharacterized PFAS present in the environment.

In 2001, the analysis of PFAS (notably PFOA and PFOS) first employed liquid chromatography-tandem mass spectrometry (LC–MS/MS) [5,12,13]. This technique has been further developed over the years to achieve the analysis of more than 50 PFAS in a single analytical run [14]. The United States Environmental Protection Agency (US EPA) has developed and validated a series of methods for PFAS using LC–MS/MS with sample preparation using solid-phase extraction [15,16]. However, some PFAS are not amenable to this technique. Lacking ionizable groups such as carboxylate or sulfonate, fluorotelomer alcohols (FTOHs) cannot be analyzed with sufficient sensitivity using LC–MS/MS. Techniques for the analysis of FTOHs include gas chromatography-mass spectrometry (GC–MS) with either electron ionization or chemical ionization [17], direct analysis using chemical ionization MS [18], and preparation with dansyl derivatives followed by LC–MS/MS [19,20]. Aldehyde and ketone-containing PFAS metabolites are often derivatized using 2,4-dinitrophenylhydrazine to enhance ionization for LC–MS/MS analysis [17,21]. While these techniques have advanced our knowledge of PFAS contamination and exposure, these are, for the most part, targeted analyte assays. As such, they only allow detection and quantitation of specified compounds for which the chemical identity is already known. While this information is useful, methods such as the total oxidizable precursor assay have determined that a significant fraction of the total PFAS present in environmental samples consist of unidentified compounds [22,23]. To better understand the full extent of PFAS contamination in the environment, it is first necessary to detect and characterize these unknown compounds.

Untargeted analysis using high-resolution mass spectrometry (HRMS) has been a key advance in detecting uncharacterized PFAS present in the environment [24,25,26,27]. The discovery of these compounds using mass spectrometry commonly involves the detection of peaks with negative mass defects resulting from the high number of F atoms present. In addition, PFAS homologues of varying chain length can be identified by peaks which differ from one another by *m/z* 49.9968 (CF_2_). As discussed in a recent review by Xiao, over 400 novel PFAS have been discovered using approaches such as these between the years 2009 and 2017 [28]. For instance, Strynar et al. first reported the presence of the PFOA-replacement, hexafluoropropylene oxide dimer acid (HFPO-DA, known by its tradename GenX), and several other novel PFAS in US surface waters using high-resolution time-of-flight mass spectrometry [24]. Since its initial discovery in surface waters, HFPO-DA has become an analyte of interest in several targeted analyses including the recent EPA Methods 537.1 and 533 for the determination of PFAS in drinking water [15,16]. In addition, untargeted methods have been used to discover a wide array of non-ionic, cationic, and zwitterionic PFAS [29,30,31,32,33]. Because these compounds would not be detected using typical analytical methods for anionic PFAS, their discovery highlights the importance of using untargeted techniques to better characterize the full extent of PFAS contamination in the environment. However, even with HRMS instruments becoming more widely available, there are many challenges associated with the discovery of novel PFAS using these techniques. For example, in complex environmental matrices, it can be a difficult to distinguish PFAS from the numerous other compounds present in a sample. Mass spectra can be filtered to remove peaks without characteristic negative mass defect [24,29], but additional selection criteria may be necessary in highly complex matrices. Even after a reasonable chemical formula is proposed from the mass spectrum, it can be difficult to confirm the structural identity of a suspect compound without analytical standards.

Regardless of whether targeted or untargeted techniques are used, there are also a number of general challenges associated with the analysis of PFAS. Of these challenges, there is perhaps none more common in a laboratory than the problem of PFAS background contamination. Because these compounds are used ubiquitously in so many products, they can be routinely detected in laboratory supplies and reagents. Consequently, great care must be taken to avoid contamination during sample preparation which could otherwise result in the false detection of certain PFAS. It is common practice to test laboratory equipment and consumable items for PFAS background levels before using them for sample preparation. Even with background from sample preparation under control, contamination can arise during analysis as PFAS are often present in the tubing, seals, and other components of analytical instruments. To mitigate this, instruments (e.g., LC–MS/MS systems) must be modified with PFAS-free parts and equipped with isolator columns that capture any remaining PFAS originating from the system and separate them from the “true” PFAS in a sample. However, it can be difficult to remove all PFAS from a system with a single technique due to the wide range of different PFAS in use.

As legacy PFAS are replaced with alternative chemistries such as short-chain PFAAs, analytical methods must expand to include a greater number of target analytes. As a result, it becomes a balancing act to analyze many compounds with a wide range of physicochemical properties including polarity, charge state, and functional group. Owing to the structural similarities between legacy and alternative PFAS such as HFPO-DA, these emerging compounds can, for the most part, be determined using the same analytical techniques used for legacy PFAS. There are, however, some analytical challenges for certain subclasses of emerging PFAS. One challenge associated with measuring HFPO-DA and other perfluoroethercarboxylic acids (PFECAs) is the fact that these compounds often exhibit poor LC–MS/MS sensitivity, which appears to be at least partially related to in-source fragmentation during electrospray ionization (ESI) [24,34,35]. However, Brase and Spink recently reported significantly enhanced sensitivity for HFPO-DA and eight other PFECAs by making facile instrument modifications [36], demonstrating that these compounds can be easily incorporated into existing methods for PFAS determination.

## 3. Environmental Fate

### 3.1. Sources of PFAS in the Environment

#### 3.1.1. Direct Sources of Legacy PFAS

The historical sources of legacy PFAS in the environment have previously been described in several other review articles [1,37,38,39]. In short, PFAS in the environment arise from both direct and indirect sources. Direct emissions, as defined by Buck et al., are the release of specific PFAS (and any unintentional byproducts or impurities) during their manufacture, use, and disposal [1]. These sources are thought to account for the vast majority of PFAS contamination in the environment [37,40]. In more developed countries, fluorochemical production plants (FPPs) have been primarily responsible for the direct release of long-chain legacy PFAS to surface water and air [37,40]. After the institution of numerous regulations as well as voluntary efforts to phase out certain legacy PFAS, release of these compounds from FPPs in many nations has gradually decreased [41,42,43,44]. However, in other countries, FPPs continue to be a significant source of legacy PFAS to the environment [45]. Other important direct emission sources are facilities using AFFFs including military bases, airports, and training facilities for firefighters [37,46]. Legacy PFAS in the runoff from these locations have been shown to infiltrate various environmental media including surface water, soil, plants, and groundwater [46,47]. Wastewater treatment plants (WWTPs), particularly those that treat industrial waste, are also significant sources of legacy PFAS in the environment. Because WWTPs are not equipped to remove or recover PFAS, the removal efficiency of these compounds throughout the treatment process is poor and they are often detected at high concentrations in WWTP effluent water [48]. In addition, because clarification and aeration tanks at many WWTPs are not covered, semi-volatile precursors and certain PFAAs can be released to the atmosphere during treatment [49]. Removal of PFAS during the WWTP process primarily occurs via adsorption to suspended solids followed by sedimentation and the formation of sewage sludge [50,51]. This sludge, however, is eventually removed from the WWTP and is often used as organic soil amendment which has been shown to contribute to the contamination of soil and agricultural crops [52]. Sewage sludge, AFFFs, textiles, and other PFAS-containing products are also routinely disposed of at incineration facilities [53,54,55,56]. It is unclear whether PFAS are released to the atmosphere in their original state during incineration; however, the release of lower-molecular-mass PFAS products has been reported [56]. Whether the release of combustion products should technically be considered as direct or indirect emission requires further consideration.

#### 3.1.2. Indirect Sources of Legacy PFAS

Most legacy PFAS of concern are stable under environmentally relevant conditions and are not known to degrade [1]. However, certain precursor compounds that can undergo transformation can serve as indirect sources of terminal PFAS to the environment. Indirect sources, as defined by Buck et al., refer only to the formation of PFAS via degradation or biotransformation of precursor compounds [1]. It should be noted that this definition differs slightly from that of Prevedouros et al., which originally classified PFAS released as impurities from the production of other PFAS to be indirect emission [37]. Although indirect emissions account for a lesser quantity of bulk PFAS in the environment [37,40], these sources play an important role in the long-range transport and global contamination of legacy PFAS. One of the more well-known examples are FTOHs—a subclass of PFAS precursors that has been studied extensively as an indirect source of PFCAs in the environment. Once released to the atmosphere, FTOHs can be oxidized to form terminal compounds including PFOA [57]. The degradation of FTOHs is thought to be one of the predominant modes of long-range, global contamination for PFOA and other PFCAs [37,57,58]. In addition to FTOHs, perfluoroalkane sulfonamido substances are another subclass of PFAS precursors that has more recently been studied as an indirect source of PFAS in the environment. The degradation of these compounds can occur biotically or abiotically and has been reported in water [59], soil [60], sludge [61], sediments [62], and mammalian cells [63]. For example, the degradation of N-ethyl perfluorooctane sulfonamido acetic acid (N-EtFOSAA) has been observed to yield PFOA by oxidation in aqueous conditions [59] and PFOS by aerobic biotransformation in soil [60].

#### 3.1.3. Landfills as Direct and Indirect Sources

As they are often used as a terminal solution for the disposal of municipal and industrial waste, landfills warrant special attention as both direct and indirect sources of PFAS to the environment. Because these facilities receive waste from numerous different inputs, PFAS burdens at landfills can be extremely high and diverse. For example, after municipal sewage and industrial wastewater is treated at WWTPs, the sewage sludge produced is often disposed of directly in landfills if it is not incinerated or used for agricultural soil amendment [54,64]. Ash produced from the incineration of sewage sludge and other PFAS-containing waste products is also routinely discarded in landfills. Depending on the temperature of incineration, PFAS and products of incomplete combustion can still be present in ash upon disposal [55]. Another major source of PFAS to landfills is the disposal of used consumer goods such as food packing materials, paper products, textiles, and carpets [64,65,66]. Leachate from these solid waste products deposited at landfills is typically collected and brought to WWTPs for treatment [64]. However, because WWTPs are not equipped for the removal of these compounds, PFAS will often be rereleased to the environment in effluent waters [48], sewage sludge [50], or air [49]. Moreover, because some facilities lack proper infrastructure and lining, leachate can be released from landfills and infiltrate neighboring groundwater supplies [67,68]. This is thought to be one of the primary modes for the direct emission of PFAS from landfills [67]. The migration of leachate from landfills is also considered to be an indirect source of PFAS to the environment due to the numerous potential pathways for the transformation of precursor compounds. For example, Allred et al. [65] and Lang et al. [66] observed the formation of degradation products such as 5:3 fluorotelomer carboxylic acid (5:3 FTCA) from the anaerobic biotransformation of precursor PFAS in model landfill reactors. In addition to the transformation of PFAS in leachate, landfills may serve as indirect sources of PFAS to the atmosphere if these compounds are not effectively captured by a gas collection system. For instance, Ahrens et al. observed significantly higher concentrations of semi-volatile precursors, namely FTOHs, in the air surrounding landfills compared with control sites [49].

#### 3.1.4. Sources of Emerging PFAS

Compared with their legacy predecessors, there is substantially less information available regarding the sources of emerging PFAS in the environment. Nonetheless, the limited studies that exist indicate the environmental presence of these emerging alternatives is still primarily the result of direct emissions from fluorochemical manufacturing. For instance, the recent detection of HFPO-DA in surface waters of the United States, the Netherlands, Germany, and China has been directly linked to fluorochemical production plants FPPs in those regions [24,25,69,70]. Similarly, 4,8-dioxa-3H-perfluorononanoic acid (DONA), which has been used as a replacement for PFOA in Germany since 2008, has mainly been detected in river water directly downstream of FPPs [71]. Another example is the industrial chemical known as F-53B, which has been used primarily in China as a replacement for PFOS in the chromium plating industry [72]. The trade name F-53B usually refers specifically to a single compound, 6:2 chlorinated polyfluoroethersulfonic acid (6:2 Cl-PFESA), but the name is often used to encompass both 6:2 Cl-PFESA and minor impurities such as the 8:2 homologue, 8:2 Cl-PFESA. The major component of F-53B, 6:2 Cl-PFESA (also sometimes referred to as 9Cl-PF3ONS [16] or C8 Cl-PFESA [73]), has been detected at the highest concentrations in close proximity to Chinese FPPs, indicating that these facilities are the primary sources of F-53B contamination [74]. Compared with levels near FPPs, environmental concentrations of emerging PFAS distant from direct emission sources remain relatively low, indicating emissions from fluorochemical manufacturing are primarily responsible for the presence of these compounds in the environment. Still, the detection of emerging alternatives such as HFPO-DA [75] in remote regions suggests that despite relatively limited production history, many of these alternatives are, or have the potential to become, global contaminants.

### 3.2. Long-Range Transport

Once released into the environment, the chemical stability of PFAS allow for their long-distance transport and persistence in the media in which they are deposited. This first became evident in 2001 when Giesy and Kannan discovered the ubiquitous presence of PFOS in wildlife from both industrialized and remote regions across the globe [12]. Shortly thereafter, Hansen et al. discovered PFOS at alarming levels in samples of human sera, along with PFOA and perfluorohexanoic acid (PFHxA) at lower concentrations [5]. Since these findings, legacy PFAS have been detected in numerous matrices and their behavior in the environment has been thoroughly summarized across several review articles [1,37,39]. Briefly, after being released from emission sources into the water and air, PFAS can undergo long-range transport to remote parts of the globe via oceanic and atmospheric currents [37,49].

**Figure 2:**
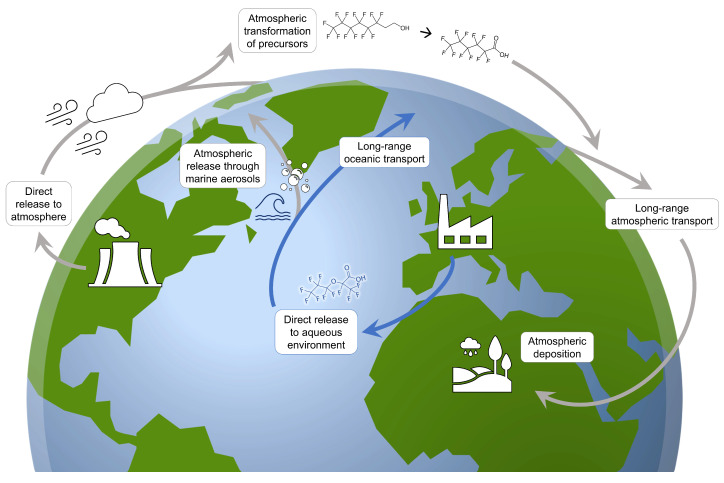
Illustration depicting some of the pathways for the long-range, global transport of PFAS in the environment.

The lifecycles of PFAS within these two modes of transport involve numerous potential pathways and interactions. In the ocean, PFAS are thought to reside primarily in the upper mixed layer due to their aqueous solubility and surfactant properties [37]. There is, however, evidence that PFAS can be carried to the deep ocean via either the downwelling or adsorption to suspended particulates followed by sedimentation [37,76]. Due to the slow turnover rates of deep ocean water and sediment, the deep ocean is thought to serve as a long-term sink for these compounds [37]. PFAS remaining in the surface mixed layer are transported long distances by oceanic currents [39] or can enter the atmosphere on marine aerosols generated by rough sea conditions and breaking waves [37,77]. PFAS present in the atmosphere can be carried far from their emission sources by wind currents and settle in the regions to which they are transported through either wet or dry deposition [58]. During this process, semi-volatile precursor compounds may undergo degradation in the atmosphere to form legacy PFAS [57,58]. The atmospheric oxidation of FTOHs and subsequent transport of terminal compounds is thought to be one of the predominant modes of global contamination for PFCAs [37,57,58]. Figure 2 summarizes some of the key steps involved in the long-range transport of PFAS through water and air.

Although the transport and distribution of legacy PFAS has been well described, there is still a lack of data regarding the long-term environmental fate of emerging compounds. As discussed in the *“Sources”* subsection, emerging PFAS such as HFPO-DA, DONA, and 6:2 Cl-PFESA have been detected at relatively high concentrations near known direct emission sources such as FPPs [24,25,69,70,71,74]. There is much less known, however, about the long-range transport of these compounds and whether this occurs predominantly in water or air. Recently, Washington et al. investigated the occurrence of several chlorinated polyfluoroethercarboxylic acids (Cl-PFECAs) in soils across the state of New Jersey, US using non-targeted MS [27]. The geographic distribution of Cl-PFECA congeners suggested that these compounds were emitted from a specific FPP to local and distant soils across the state via atmospheric transport. In addition, one congener was detected in a soil sample from New Hampshire, US (roughly along the same sampling transect), but further investigation is needed to confirm whether this was released from the same FPP [27]. In another recent study, Joerss et al. investigated the distribution of HFPO-DA and numerous legacy PFAS in ocean water samples collected between the European coastline and the Fram Strait, located south of the Arctic Ocean [75]. The concentration gradient of HFPO-DA in ocean water decreased with increasing distance from the European coastline, suggesting that emissions from European waters are likely the primary source of this compound. However, mass transport profiles for other PFAS suggested that long-range atmospheric deposition contributes significantly to legacy PFAS in Arctic outflow [75]. This study demonstrated for the first time the long-range transport of HFPO-DA to remote regions.

### 3.3. Concentrations in Biota

The propensity for PFAS to undergo long-range transport and to persist in the media in which they are deposited has led to their ubiquitous detection in numerous environmental matrices across the globe [4,12,78]. After having been transported to a particular region, PFAS present in the environmental media (e.g., water, soil, air) can be taken up and concentrated by local flora and fauna [4,79,80,81]. As discussed above, the bioconcentration of PFAS in biota was first demonstrated by Giesy and Kannan in 2001, after they discovered PFOS in wildlife from remote regions across the globe as well as in industrialized areas [12]. In general, greater PFAS concentrations are observed in higher-trophic-level species, indicating that these compounds undergo biomagnification in the food chain [82]. The occurrence of PFAS in wildlife has been well characterized for many legacy compounds since the original findings of Giesy and Kannan [28,78,83,84,85]; however, far less data are available regarding the concentrations of emerging PFAS in biota. Studies that have reported concentrations of emerging PFAS in wildlife, to our knowledge, have focused on a very limited number of compounds. Relatively low concentrations of the PFOA substitute, DONA, have been reported in grass [71] and deer [86] from Germany by the Bavarian Environment Agency (LfU). In a more recent study, DONA was also detected in locusts from China, but only in a single pooled sample collected at one site [87]. The most frequently detected compound, 6:2 Cl-PFESA, has been measured in Arctic mammals [83], fish [88], amphibians [89], and captive tigers [90]. The greater detection frequency of 6:2 Cl-PFESA is likely due to the fact that it has been used in China for over 30 years [72], although its use has been restricted to China. It is thus unclear whether 6:2 Cl-PFESA should be classified as an “emerging” compound. More recently, the PFOA substitute, HFPO-DA, has been detected in plants [34] and fish [79,91]. In addition, the trimer acid of HFPO, hexafluoropropylene oxide trimer acid (HFPO-TA) has been detected in fish and amphibians [79,89]. Table 1 shows concentrations of these emerging PFAS in biota from different regions across the globe.

## 4. Human Exposure and Biomonitoring

Measurable levels of PFAS in humans can be linked to both occupational and non-occupational exposure. This has been extensively reviewed by Sunderland et al. in 2019 [92]. Many industries can contribute to occupational exposure to PFAS including fluorochemical production workers [93,94], firefighters [95,96,97,98,99,100], ski wax technicians [101,102], and metal platers [73]. Non-occupational exposure pathways of PFAS have been linked to PFAS contaminated drinking water and food as well as dust inhalation [92,103]. Recent reviews by Domingo and Nadal have focused on the two major exposure routes of PFAS at a global scale via drinking water and dietary intake [104,105].

Human biomonitoring is used to assess the exposure of a population to environmental chemicals and toxic substances. National biomonitoring programs have been established by many countries including the United States (National Health and Nutrition Examination Survey; NHANES), Canada (Canadian Health Measures Survey), Germany (Human Biomonitoring Commission of the Federal Environmental Agency), and France (French Ministries of Health and Environment). In addition, the European Union established the European Human Biomonitoring Initiative, which is a collaboration among 28 countries, the European Environment Agency, and the European Commission.

The majority of early PFAS biomonitoring studies primarily focused on serum PFOA and PFOS. Many of these studies have been included in previous review articles [92,106,107,108] and will only be described briefly here. Since the mid-2000s, there has been an observed decline in PFOA and PFOS levels in humans that correlates with the production phaseout of these compounds. In the US, serum PFOA and PFOS levels increased over the years 1974 through 1989 before decreasing in the early 2000s [93,109,110,111,112]. Canada has seen a similar trend based on biomonitoring projects conducted by the Human Biomonitoring of Environmental Chemicals in Canada program, noting a decline in serum PFOA and PFOS over the years 2007 through 2017 [113]. In studies from Japan, an increase in serum PFOA between 1983 and 1999 was reported [114,115]. A biomonitoring study out of Shenyang, China showed an increase in PFOA and PFOS exposure between 1987 and 2002 [116]. Similar trends have also been seen in European countries. A study involving 57 pooled serum samples collected from men in Norway between the ages 40 and 50 showed an increase in PFOS, PFOA, and perfluoroheptanesulfonic acid (PFHpS) between 1977 and the 1990s followed by a decline starting in the early 2000s [117].

Numerous studies have been conducted with high-exposure populations to assess the half-lives of PFAS in humans. In general, longer-chain PFAS are observed to have longer half-lives. This has been attributed to renal elimination mechanisms. Han et al. showed that shorter-chain PFAS have lower binding affinities for renal organic anion transport proteins which, along with higher water solubility of shorter-chain molecules, play a large role in PFAS elimination [118]. The half-lives of PFAS tend to be shorter in women compared with men. This is attributed to elimination from the body via menstruation, placental transfer during pregnancy, and lactational transfer via breastfeeding [119,120]. Additionally, differences in the half-lives among PFCAs and PFSAs have been observed. For both short- and long-chain compounds, PFSAs tend to have longer half-lives than PFCAs with the same number of carbons (e.g., PFOS and PFOA) [93,121,122,123]. However, it should be noted that, because the carbon atom of the carboxylic acid functionality is used in the numbering and naming of PFCAs, many groups compare PFCAs with PFSAs of the same *perfluorinated* carbon chain length (e.g., PFOA and PFHpS) to better characterize the role of different acid functionalities [124,125,126,127]. Even so, PFSAs have been observed to have longer half-lives compared with PFCAs of the same perfluorinated carbon chain length [126], suggesting differences between the acid functionalities influences elimination. Most half-life studies are conducted by measuring changes in blood PFAS concentrations over time. Few studies have estimated the half-lives of short-chain PFAS due to the low levels detected in blood and their fast elimination rates following exposure. Some urinary excretion studies have been performed to estimate half-lives for these compounds. Table 2 shows half-lives of selected PFAS by country.

Based on the physiochemical properties of PFAS, many biomonitoring studies are conducted using serum or plasma. Additional studies have been conducted using whole blood, dried blood spots, milk, urine, hair, and even nails to assess human exposure. Different techniques and tissue distributions have been reported in a recent review by Jian [106]. Due to the worldwide distribution and environmental persistence of legacy PFAS such as PFOA and PFOS, there have been substantial efforts for the biomonitoring of these compounds. However, fewer studies have focused on emerging alternatives such as PFECAs and perfluoroethersulfonic acids (PFESAs) as a result of their relatively short production history.

In North Carolina, US drinking water from the Cape Fear River was determined to contain HFPO-DA at low part-per-billion levels. Citizens in the surrounding area were asked to participate in a biomonitoring study to determine human exposure [131]. This study investigated the concentrations of HFPO-DA, DONA, 6:2 Cl-PFESA, and 14 legacy PFAS. HFPO-DA, DONA and 6:2 Cl-PFESA were not detected (LOD 0.1 μg/L) in any of the study participants (*n* = 30). The authors suspect this is because participants switched to bottled water months before sample collection and that the half-lives of these emerging compounds in humans are likely short. Successful biomonitoring of these emerging compounds in serum must be conducted immediately after exposure. Urine samples may be more practical for biomonitoring studies involving these compounds, since they are expected to have shorter half-lives and may be efficiently eliminated in urine [132]. Another study in the US analyzed 2682 archived urine samples from participants aged 12 and older from the 2013–2014 NHANES [133]. HFPO-DA was detected in 1.2% of the samples, with urine concentrations ranging from 0.07 to 0.3 μg/L, while 6:2 Cl-PFESA and DONA were not detected in any samples.

Studies from Germany report on the analysis of serum samples for HFPO-DA, 6:2 Cl-PFESA, 8:2 Cl-PFESA, DONA, 7H-perfluoroheptanoic acid (7H-PFHpA), perfluoroethylcyclohexanesulfonic acid (PFECHS), fluorotelomer sulfonic acids (FTSs), and fluorotelomer phosphate diesters (diPAPs) [134,135]. Analyses of plasma samples collected between 2009 and 2019 and archived in the German Environmental Specimen Bank produced a 1% detection frequency for 8:2 FTS; however, no other emerging PFAS were detected [134]. In a separate study, Fromme et al. analyzed 396 plasma samples collected between 2009 and 2016 from blood donors living in South Germany [135]. DONA was detected above the limit of quantitation (LOQ > 0.2 μg/L) in approximately 6.5% of the samples with a maximum concentration of 14.4 μg/L [135].

Emerging PFAS of concern in China include compounds and byproducts related to the metal plating industry, such as 6:2 Cl-PFESA. In a study by Shi et al., 6:2, 8:2, and 10:2 Cl-PFESA serum concentrations were determined in the general population (*n* = 8), high fish consumers (*n* = 45), and metal plating workers (*n* = 19) [73]. Mean total Cl-PFESA concentrations and corresponding ranges were 4.20 ng/mL (1.91–6.01) for the general population, 102 ng/mL (1.97–360) for high fish consumers, and 941 ng/mL (2.42–5127) for metal plating workers. In a separate study, Chen et al. examined the correlation between maternal and umbilical serum levels of two Cl-PFESAs and found that 6:2 and 8:2 Cl-PFESA could be efficiently transported across the placenta [136]. The authors hypothesized that 8:2 Cl-PFESA was transported across the placenta to a greater extent due to its higher hydrophobicity and lower plasma protein binding affinity.

## 5. Metabolism of PFAS

Due to the unusual strength of C–F bonds, most PFAS are highly resistant to metabolism. For example, PFOA and PFOS are considered essentially non-metabolizable and are eliminated in urine and bile in their original state. However, certain subclasses of PFAS compounds and synthetic precursors can undergo limited metabolism. Of note in this regard are FTOHs—the metabolism of which has been studied extensively in rodents [137]. The telomeric carbons, which have C–H rather than C–F bonds, are the susceptible to oxidative metabolism that leads to the formation of FTCAs and PFCAs including PFOA [137]. A somewhat unique group of individuals that has been investigated with respect to the potential for human metabolism of FTOHs is that of ski wax technicians. These individuals, who are occupationally exposed to vapor-phase FTOHs [138,139], show patterns of metabolites consistent with the metabolism of FTOHs to FTCAs and PFCAs as have been observed in toxicokinetic studies in rodents [137]. Based on experimental studies, there are additional precursor PFAS that undergo limited metabolism. In rats, perfluoroalkyl phosphate esters (PAPs) are converted to PFCAs [140] and perfluorooctane sulfonamide (FOSA) is metabolized to PFOS [141]. Comparable metabolism of these compounds may occur in humans. In rats, HFPO-DA appears to be eliminated without metabolism [142].

## 6. Health Impacts of PFAS

### 6.1. Animal Toxicology Studies

Most of the initial toxicology studies of PFOA in experimental animals dating from 1965 through 1983 are in the form of unpublished reports of the DuPont Company. Results of many of the standard toxicity tests at DuPont including acute oral toxicity, acute dermal toxicity, acute inhalation toxicity, skin and eye irritation, dermal irritation and sensitization, eye irritation, and the corresponding subchronic toxicity studies have been summarized by Kennedy et al. [143]. Acute oral toxicity of PFOA was characterized as moderate, with LD_50_s of 178 mg/kg in male and 217 mg/kg in female guinea pigs, and 470 mg/kg in male and 482 mg/kg in female rats [143]. Additional studies of PFOA in rodents showed impaired mammary gland development, decreased litter weight, decreased pup survival, neurodevelopmental effects, and impaired immune response in studies using dosing regimens of 0.01 to 5 mg/kg/day; PFOA doses in the range of 10 to 20 mg/kg/day resulted in hepatocellular necrosis [144]. PFOS has been shown to have similar toxic effects in rodents, and PFOS appears to be particularly potent in eliciting immunomodulatory effects [144,145,146,147,148,149]. PFOS exposure caused marked decreases in several immunologic parameters including natural killer cell activity and the number of CD4^+^ cells in C57BL/6 mice [147]. The lowest observed adverse effect level for the T cell-dependent antibody response (TDAR) for the production of sheep red blood cell-specific IgM production, which is considered the most sensitive measure of immunomodulation, was 3.75 mg/kg/day for PFOA in C57BL/6 female mice [150].

Not only do PFAS appear to be toxic in terrestrial mammals, there is significant evidence that PFAS accumulate and have toxic effects in aquatic mammals, birds, fish, amphibians, reptiles, and numerous invertebrates [91,148,151,152,153,154,155]. In detailed studies of the Great Lakes region of the US, PFAS have been reported in various trophic levels of the aquatic food chain with concentrations of PFAS in benthic invertebrates 1000-fold greater that the surrounding water and biomagnification factors of 5–10 between the livers of salmon and the livers of eagles and mink [82]. In numerous investigations, significant associations among infectious diseases and elevated PFAS levels in aquatic mammals including Atlantic bottlenose dolphins [156] and sea otters [157] have been reported that may be indicative of immunodeficiency in these animals. Studies with a resident population of heterogeneous, free-ranging Atlantic bottlenose dolphins in the Charleston, South Carolina, US region suggest that PFOS directly dysregulates the dolphin cellular immune system. Baseline PFOS concentrations were associated with significantly increased proliferation CD4^+^ and CD8^+^ T cells. Based on these and other findings, Soloff et al. [158] concluded that PFOS exposure induces the production of proinflammatory interferon-γ, but not immunoregulatory interleukin-4 in T cells, and this may establish a state of chronic immune cell activation that is known to be associated with susceptibility to disease.

The overwhelming majority of the toxicological studies on PFAS have investigated specific legacy compounds such as PFOA and PFOS; however, in recent years, these compounds have been replaced by emerging PFAS subclasses including PFECAs in industrial processes. There are public health and environmental concerns regarding the replacement compounds, as recent studies have shown that PFECAs and a novel PFESA (referred to as Nafion byproduct 2) are now appearing in drinking water sources as well as in juvenile seabirds [155] and striped bass [91] from Atlantic coastal regions of the US. There is limited toxicological data on most PFECAs; however, HFPO-DA, which was developed as the primary replacement for PFOA, has been investigated in a number of studies. The initial 28 day oral gavage studies in mice and rats and 90 day oral gavage studies in rats of HFPO-DA were performed between 2008 and 2009 by WIL Research Laboratories (now Charles River Laboratories) and DuPont, and are summarized by Thompson et al. [159]. Further studies of the liver sections from the 90 day oral gavage study of HFPO-DA in male and female mice [159], which include gene expression studies, are reported by Chappell et al. [160]. The chronic toxicity and carcinogenicity of HFPO-DA were evaluated in a 2 year oral dosing study in Sprague–Dawley rats [161]. Acute oral LD_50_ values were 1750 mg/kg for male rats and 3129 mg/kg for female rats. The more rapid clearance of HFPO-DA in comparison with legacy PFAS is thought to be at least partially responsible for the reduced toxicity and bioaccumulation of HFPO-DA [159].

Results from the 2 year oral dosing study indicated that HFPO-DA exposure in rats caused significantly increased liver/body weight ratios at the 50 mg/kg dose in males and at 500 mg/kg in females at the 12 month interim sacrifice [161]. Clinical pathology indicative of liver injury was present in exposed animals, with no observed adverse effect levels falling between 1 and 50 mg/kg for males and between 50 and 500 mg/kg for females [161]. Numerous PFAS are peroxisome proliferators and activate peroxisome proliferator-activated receptor α (PPARα) [144]; the propensity of HFPO-DA to elicit PPARα-mediated gene expression was therefore investigated. The enhanced expression of gene sets specifically regulated by PPARα was observed, which include genes encoding numerous enzymes involved in lipid metabolism [160]. There is significant literature on the differences between rodent and human PPARα and their respective roles in gene expression, metabolism, and toxicology, which will be discussed in succeeding sections of this review. There is significant evidence that PPARα-regulated pathways observed in rodents are not predictive of or relevant to human toxicology.

The immunotoxicity of HFPO-DA and the potential detrimental effects elicited during gestational exposure to HFPO-DA have also been investigated. To evaluate the immunotoxicity of HFPO-DA, TDAR and lymphocyte proliferation were determined in C57BL/6 mice of both sexes gavaged with 0, 1, 10, or 100 mg/kg/day of HFPO-DA for 28 days [162]. HFPO-DA exposure at 10 and 100 mg/kg/day suppressed TDAR in mice of both sexes; T lymphocytes were elevated in males exposed to 100 mg/kg/day. These effects are similar to those of PFOA but show a reduced potency of HFPO-DA in comparison with PFOA. To investigate adverse effects during gestation, pregnant CD-1 mice [163] and Sprague–Dawley rats [164,165] were dosed with HFPO-DA by oral gavage. Pregnant CD-1 mice exposed to HFPO-DA at 2 and 5 mg/kg/day showed increased maternal liver weights, increased placental weights, and elevated embryo–placenta weight ratios relative to controls. A greater incidence of placental abnormalities was also observed in HFPO-DA-exposed CD-1 mice. In the studies with Sprague–Dawley rats, HFPO-DA-exposed rats during gestational days 14 through 18 showed higher maternal liver weights and reduced maternal thyroid hormone levels, and elevated levels of PPAR-regulated gene expression were observed in both the fetal and maternal livers of HFPO-DA-exposed rats [164]. In subsequent studies with variable dosing (from 1 to 125 mg/kg/day) from gestational day 8 through post-natal day 2, dose-responsive increases in neonatal mortality, decreases in pup birth weight, and increases in pup liver weight were observed [165].

### 6.2. Non-Cancer-Related Health Impacts in Humans

While numerous studies have detailed toxic effects of PFAS in an array of animal species, the relevance of many of these effects to human health has been questioned. The potential effects of PFAS on human health have been investigated in depth in numerous studies over the past several decades. Recently, the immense data sets on health effects of PFAS have been compiled, subjected to review by panels of experts, and the summaries have been made available by several working groups [144,166,167]. Several extensive literature reviews on the health effects of PFAS in highly exposed populations and those only exposed to background levels are also available [92,168,169]. Our goal here is to summarize the major findings from these national and international policy statements, extensive reviews, recent studies, and other important data resources.

By the early 2000s, it became clear that the environmental contamination of PFAS was an issue at the global scale resulting in significant human exposure. A major advance in our understanding of the human health effects of PFOA came in the studies focused on the mid-Ohio Valley region in the US, specifically, in the vicinity of DuPont’s Washington Works plant near Parkersburg, West Virginia. The plant manufactured Teflon for many years, and evidence indicates that in the approximate years of 1984 through 2004, discharges from the plant contaminated the Ohio River, the source of public drinking water for many residents of Ohio and West Virginia living nearby the Washington Works facility. Spurred by legal action, a group of epidemiologists known as the C8 Science Panel was established to investigate probable links between PFOA exposure and human disease. In the period 2005–2006, the C8 Health Project group was created and enrolled 69,030 participants for 11 distinct epidemiological studies. The studies incorporated known rates of plant emissions, modeling of PFOA transport, and measurements of serum PFOA to assess exposure. Longitudinal studies over 6 years identified probable links to kidney and testicular cancer, pregnancy-induced hypertension, thyroid disease, high serum cholesterol, and ulcerative colitis [169].

In 2018, the Agency for Toxic Substances and Disease Registry (ATSDR) of the US Centers for Disease Control and Prevention, Department of Health and Human Services released its Toxicological Profile for Perfluoroalkyls for public comment [144]. This toxicological profile considered available toxicologic and epidemiologic data on 14 legacy PFAS: perfluorobutanoic acid (PFBA), PFHxA, perfluoroheptanoic acid (PFHpA), PFOA, perfluorononanoic acid (PFNA), perfluorodecanoic acid (PFDA), perfluoroundecanoic acid (PFUdA), perfluorobutanesulfonic acid (PFBS), perfluorohexanesulfonic acid (PFHxS), PFOS, perfluorododecanoic acid (PFDoA), FOSA, N-methyl perfluorooctane sulfonamido acetic acid (N-MeFOSAA), and N-EtFOSAA. This document considered both epidemiologic data and animal studies. For the epidemiologic studies, three types of populations were considered: (1) those who were occupationally exposed through employment in a PFAS production facility; (2) members of the community who were exposed to PFAS by living in the vicinity of a PFAS production facility; and (3) those who were exposed to background levels of PFAS. It should be noted that these three groups differ markedly in their PFAS exposure levels when assessed by serum levels of PFAS. Girardi and Merler reported serum PFOA concentrations as high as 91,900 ng/mL in occupationally exposed individuals [170], while a range of 2600–5200 ng/mL for serum PFOA was considered the definite exposure group by Lundin et al. [171]. In the C8 Health Project, the geometric mean serum PFOA concentration for those exposed in the mid-Ohio valley community was 32.9 ng/mL [172], whereas the geometric mean serum PFOA concentration in the US general population in the period 2005–2006 was 3.9 ng/mL [173]. Based on review of epidemiology and animal studies, it was concluded that there is evidence of hepatic, cardiovascular, immune, reproductive, and developmental effects associated with PFAS exposure [144].

Of the potential human health outcomes for PFOA and PFOS that have been identified, there is perhaps the strongest evidence for immunotoxic effects that are consistent with studies of experimental animals and observations in wildlife. In a study of a birth cohort in the Faroe Islands, Grandjean et al. reported changes in antibody concentrations for children aged 5–7 in association with serum PFAS [174]. In this study, a 2-fold increase in maternal serum PFOS was associated with a 39% decrease in the child’s diphtheria antibodies at age 5 (95% CI: −55% to −17%, *n* = 510), but a similar significant association was not observed for PFOA. A 2-fold increase in the child’s serum PFOS at age 5 was associated with a 28% decrease in diphtheria antibodies at age 7 (95% CI: −46% to −3%, *n* = 408) and a 29% decrease in tetanus antibodies at age 5 (95% CI: −46% to −6%, *n* = 440). A 2-fold increase in the child’s serum PFOA at age 5 was associated with a 25% decrease in diphtheria antibodies (95% CI: −43% to −2%, *n* = 408) and a 36% decrease in tetanus antibodies (95% CI: −52% to −14%, *n* = 408) at age 7. The Norwegian Mother and Child Cohort Study reached similar findings regarding anti-vaccine antibodies, reporting significant inverse associations between the levels of anti-rubella antibodies and four PFAS (PFOA, PFOS, PFHxS, and PFNA) in the serum of the children (*n* = 50) at age 3 years [175]. In a study of adults in mid-Ohio, US exposed to PFOA through contaminated drinking water, an association was observed with reduced antibody titer response to the A/H3N2 influenza virus, suggesting that an immunotoxic effect of PFAS on humoral immunity is not confined to children [176]. In this study, an odds ratio of 0.34 (95% CI: 0.14–0.83, *p* = 0.02, *n* = 102) was reported for decreased likelihood of seroprotection from A/H3N2 in association with serum PFOA levels between 13.8 and 31.5 ng/mL (2nd quartile). An extensive review of these studies and numerous others led the National Toxicology Program of the US Department of Health and Human Services to conclude that PFOA and PFOS are presumed to be immune hazards in humans [177].

As first reported by the C8 Science Panel [172], high serum cholesterol associated with exposures to both PFOA and PFOS has been observed in several cohorts [178,179] and is considered the key finding for these compounds by the European Food Safety Authority (EFSA) [166]. Changes in lipid profiles, glucose homeostasis, and serum proteins that were also observed in association with elevated cholesterol led Liu et al. to suggest an association between PFOA exposure and metabolic syndrome [179]. Based on animal studies and human epidemiology, there is also concern that PFAS exposure is associated with neurotoxicity and delays in neurodevelopment [180,181,182,183,184], thyroid hormone disruption [185,186,187,188], altered kidney function [187,189], changes in reproductive health [190,191,192,193], and disruption of bone-cell differentiation [194]. Recent studies report reduced bone density in relation to PFAS exposure [195,196,197,198]. Analyses of dry bone and bone marrow from cadavers have shown that PFNA, but not PFOA or PFOS, was present in bone [194]. Another recent study provides evidence that PFOA disrupts vitamin D activity through binding to the vitamin D receptor, which may indicate a mechanism involved in the effects of PFOA on bone mineralization [199].

One challenge in the determination of the health effects of PFOA and PFOS is the decrease in exposure to these compounds over time due to production phaseout. The clearance rates of PFAS, which further complicate cause-and-effect relationships in longitudinal studies, are becoming well established [123]. The increases in environmental concentrations of numerous replacement compounds and confounding co-exposures to other contaminants have also complicated toxicological evaluations of PFAS. While recent investigations have included emerging PFAS such as HFPO-DA [162,163,184,200,201], the vast majority of studies have focused primarily on legacy PFAS, and the findings from these studies may not be directly relatable to emerging replacement compounds.

### 6.3. PFAS and Human Cancer

There have been numerous studies in which the potential association between PFAS exposure and cancer incidence has been investigated. Two main classes of study cohorts have been workers in PFAS production facilities [170,171,202] and community members who were exposed through the contamination of public water supplies [203,204]. In studies of the C8 Health Project group described in the previous section, increased incidence of testicular, kidney, prostate, ovarian, and non-Hodgkin lymphoma cancers were reported to be associated with PFOA exposure in the mid-Ohio valley [204]; however, not all associations were statistically significant. For kidney cancer, adjusted odds ratios of 2.0 (95% CI: 1.3–3.2, *n* = 22) and 2.0 (95% CI: 1.0–3.9, *n* = 9) were reported in the “high” (serum PFOA 30.8–109 ng/mL) and “very high” (serum PFOA 110–655 ng/mL) exposure groups, respectively. Adjusted odds ratios for non-Hodgkin lymphoma in the “very high” and “medium” (serum PFOA 12.9–30.7 ng/mL) were 1.8 (95% CI: 1.0–3.4, *n* = 11) and 1.5 (95% CI: 1.0–2.2, *n* = 28), respectively. The authors described several limitations of the data such as relatively weak measures of association, inconsistent associations across different exposure levels, and general imprecision due to the small number of cases. In a separate review, Chang et al. assert that an association between PFOA or PFOS exposure and the incidence of any cancer has not been established [205]. The discordance between cancer types and incidence among those exposed to very high levels of PFOS in the occupational setting at the Washington Works facility and those who were exposed to PFOA in the mid-Ohio valley community through contaminated drinking water was noted [205]. The relevance of studies in rodents showing increased incidence of benign tumors in rats to the prediction of PFAS carcinogenicity in humans was also questioned [205].

If PFAS are carcinogenic, the question of how, mechanistically, these compounds could initiate or promote cancer [206,207] becomes paramount. To address the possible roles of PFAS in carcinogenesis, Temkin and colleagues [208] applied the Key Characteristics of Carcinogens approach [209,210] to the existing data for 26 PFAS. The use of these characteristics provides an organizational approach to the hazard-based assessment of suspected carcinogens, which then prompts further epidemiologic studies and low-dose exposure studies in animals to evaluate carcinogenic potency. In this model, the 10 Key Characteristics of Carcinogens are that they: (1) are electrophilic or can be metabolically activated; (2) are genotoxic; (3) alter DNA repair or cause genomic instability; (4) induce epigenetic alterations; (5) induce oxidative stress; (6) induce chronic inflammation; (7) are immunosuppressive; (8) modulate receptor-mediated effects; (9) cause cell immortalization; and (10) alter cell proliferation, cell death or nutrient supply. Carcinogens may act through one or more of these processes. Their assessment was that PFAS have several of these characteristics [208].

There is no evidence that PFOA, PFOS, or other PFAS that have been studied can be metabolically activated to reactive intermediates. As noted in a previous section, PFOA and PFOS are eliminated from humans in urine and bile in their original state. While some PFAS such as FTOHs, PAPs, and perfluoroalkane sulfonamide substances likely undergo limited metabolism, there is no indication that reactive intermediates of concern would be formed. Genotoxicity and mutagenicity assays for PFOA, PFOS, and a number of other legacy PFAS have been negative [143,144,166,211] and there have been no reports of alterations of DNA repair or genomic instability associated with PFAS exposure. PFAS exposure is known to induce oxidative stress [212,213,214] and this effect is postulated to have a role in PFAS-induced carcinogenesis. In their review, Temkin et al. [208] concluded that there is insufficient evidence that PFAS induce chronic inflammation. As noted earlier, there is significant evidence that PFAS are immunotoxic [177]; however, the potential role of an immunosuppressive effect of PFAS in carcinogenesis has not been established. There is insufficient experimental evidence to determine the potential effects of PFAS exposure on genomic stability or cellular immortalization. While studies have investigated the effect of PFAS exposure on epigenetic changes in DNA methylation [215], the significance of the observed changes regarding carcinogenesis is not clear.

There is an abundance of evidence that PFAS bind to and activate nuclear receptors in mammalian cells that regulate metabolism and alter cell proliferation. It has long been known that PFOA and other PFAS bind to and activate PPARα [216,217], a member of the PPAR family of nuclear receptors that regulate receptors influencing various aspects of metabolism, energy homeostasis, development, and differentiation. The endogenous ligands for PPARα appear to be fatty acids and eicosanoids, although a variety of xenobiotics including environmental contaminants (e.g., phthalates [216,218]) and pharmaceuticals which target the receptor [219,220] activate PPARα. The laboratory evidence that persistent activation of PPARα by PFOA or other ligands leads to an elevated incidence of hepatocellular tumors in rats and mice is significant [143,216,221,222]. Recently, a study involving 120 factory workers showed elevated incidence of liver cancer following exposure to high levels of PFOA [170]. Aside from this study, there is little evidence that the liver is a major target organ for potential PFAS-induced carcinogenesis in humans. Moreover, there is a widely held view that the increased incidence of hepatocellular adenomas and carcinomas in rats and mice in response to PPARα activation is not relevant to carcinogenesis in humans [143,205,223,224].

The apparent dichotomy between the carcinogenic effects of peroxisome proliferators (e.g., PPARα agonists) in rodents versus humans has been noted for some time [218,225]. Compounds such as fibrates, which cause massive hepatomegaly, peroxisome proliferation, and liver tumors in rats [225] are safe and effective therapeutic agents for the treatment of dyslipidemia and cardiovascular diseases [220]. To investigate the underlying biochemical and genetic reasons for the species differences in carcinogenicity of PFAS and other PPARα ligands, studies were conducted using gene knock-out [226,227] and transgenic mice [217] together with gene expression profiling [228] and traditional methods of biochemical toxicology. It was observed that following exposure to a peroxisome proliferator, clofibrate or Wy-14,643, murine PPARα (mPPARα) knockout mice did not display the typical responses of hepatomegaly, peroxisome proliferation, and transcriptional activation of target genes as were observed in wild-type mice [226]. When the hepatic gene expression profiles of wild-type and mPPARα-null mice were exposed to PFOA, PFOS, Wy-14,643, or control vehicle, it was found that the majority of the gene expression changes in response to PFOA and PFOS exposure could be attributed to activation of mPPARα [228]. It was estimated that approximately 11 to 24% of the changes in gene expression in response to PFOA and PFOS exposure were independent of mPPARα but appeared to involve activation of the constitutive activated receptor, PPARγ, or estrogen receptor α.

The studies with mPPARα-null mice provided strong evidence for the role of PPARα in the physiologic/toxic responses to PFAS. Transgenic experiments were then conducted to determine whether the basis for the differences in carcinogenicity of PPARα agonists in mice and humans might lie in the differences between human PPARα (hPPARα) and mPPARα. Mice humanized for PPARα, meaning that they expressed hPPARα in their livers rather than mPPARα, were generated [229]. When these hPPARα transgenic mice were exposed to Wy-14643, elevations in fatty acid metabolizing enzymes and reductions in serum triglycerides were observed; however, hepatocellular proliferation and hepatomegaly were observed in wild-type mice but not in hPPARα transgenic mice. Cell-cycle control genes including c-*myc*, cyclin D1, and cyclic-dependent kinases 1 and 4 were upregulated in wild-type mice but not in hPPARα transgenic mice [229], and these expression changes occur concomitant with the PPARα-dependent increase in hepatocellular carcinomas in Wy-14,643-treated mice [230]. Further studies with mPPARα-null mice offered a potential mechanism for the mPPARα-mediated upregulation of c-*myc* involving the let-7c microRNA [231], which occurs in wild-type mice but not in hPPARα transgenic mice [232,233]. These studies with mouse models, when taken together with the observed differences in the carcinogenesis of fibrates and other PPARα agonists in humans and rodents, have led numerous investigators to question the efficacy of the rodent hepatocarcinoma model for predicting the cancer risk of PFAS exposure in humans [224].

In addition to liver cancer, there is significant concern that PFAS may cause cancer in extrahepatic tissues. The potential roles of PPARα-dependent and/or PPARα-independent pathways in putative PFAS-induced carcinogenesis outside the liver and the possible mechanisms that may be involved are far less clear. As noted in our review of various epidemiologic studies, testicular, kidney, and prostate cancers are among those that have been associated with PFAS exposure. In studies with rats, increases in hyperplasia and adenomas of Leydig cells of the testes in PFOA-treated rats were observed [221,222]. Since the proliferation of peroxisomes, as assessed using peroxisomal lipid β oxidation activity, did not occur in Leydig cells of the testes of rats exposed to PFOA or Wy-14643, the authors proposed that these tumors are formed by a mechanism that is independent of PPARα [221]. Numerous questions remain regarding the possible causation of various extrahepatic cancers in humans by PFAS. While PFAS were found to have no ability to activate estrogen or androgen receptors or modulate steroidal activity [234], some studies have reported an association between PFAS exposure and breast cancer in the Inuit population of Greenland [235,236]. In 2011, Bonefeld-Jørgensen et al. reported an adjusted odds ratio of 1.03 (95% CI: 1.00–1.07, *n* = 9) for breast cancer in association with PFOS exposure (median serum PFOS 45.6 ng/mL), but the association was weak and the sample size small [235]. In a 2017 study using a larger sample from the same Inuit population, Wielsøe et al. reported adjusted odds ratios of 5.50 (95% CI: 2.19–13.84, *n* = 44) and 2.64 (95% CI: 1.17–5.97, *n* = 37) for breast cancer in association with exposure to PFOS (median serum PFOS 35.5 ng/mL) and PFOA (median serum PFOA 2.08 ng/mL), respectively [236]. In contrast, Hurley et al. [237], in a nested case–control study of the California Teachers Study, observed no evidence of an association of exposure to PFOA or several other PFAS with breast cancer. A difference between the Inuit and California Teachers studies is that, in addition to PFAS exposure, the Inuit population also showed higher concentrations of polychlorinated biphenyls and several other persistent organic pollutants that were associated with breast cancer [235,236]. Potential additive or synergistic effects among combined environmental or occupational exposures further complicate the determination of carcinogenic effects in the human population.

Given the uncertainties that remain regarding the potential carcinogenicity of PFOA and other PFAS, the International Agency for Research on Cancer [238] concluded that PFOA is possibly carcinogenic to humans and assigned the compound to Group 2B. The US EPA concluded that there is suggestive evidence of the carcinogenic potential of both PFOA [167] and PFOS [239] in humans. The EPA considered the association of testicular cancer with PFOA in highly exposed workers and the increase in Leydig cell tumors in the testes of PFOA-exposed rats observed in the study by Butenhoff et al. [222] to be of sufficient quality to allow estimations of dose-response for a potential carcinogenic effect in humans. Using these data, it was estimated that a drinking water concentration of 0.5 µg/L PFOA would increase the risk of testicular cancer by one in a million [167]. The suggestive evidence of the carcinogenic potential of PFOS was based on the study by Thomford [240], which showed increased liver and thyroid adenomas in PFOS-exposed rats. The EPA has issued lifetime drinking water health advisories of 0.07 µg/L for both PFOS and PFOS.

## 7. Conclusions and Future Directions

This review summarizes pertinent literature of the last two decades regarding the production history, analytical techniques, and environmental fate for both legacy and emerging PFAS. In addition, relevant studies in biomonitoring, epidemiology, and toxicology are examined to investigate the potential human health outcomes following exposure to these compounds. While a substantial amount of data is available on these topics for legacy compounds, further research on emerging PFAS is essential to better characterize their behavior in the environment and to understand what risks they may pose to human health. Currently, it is unclear whether any emerging PFAS are degraded under environmentally relevant conditions or whether they can be metabolized by humans. Furthermore, the full extent of environmental contamination for these emerging compounds remains unclear. Developing new, or modifying existing, methods for the determination of emerging PFAS in drinking water and human matrices will be of particular importance for assessing population exposure to these compounds.

It is unlikely, however, that these emerging compounds alone can account for discrepancies between total organic fluorine [241] and concentrations of known PFAS measured in humans. There are likely hundreds, if not thousands, of unknown PFAS present in the environment and in humans that have yet to be characterized. Although much research progress has been made for both legacy and emerging compounds, our current knowledge regarding the full extent of PFAS contamination likely represents only the tip of the iceberg. For this reason, the development of untargeted methods for determining unknown PFAS present in environmental and human matrices remains crucial.

## Figures and Tables

**Table 1:** Concentrations of emerging PFAS detected in biota from various locations across the globe. Note that PFAS concentrations in additional matrices are available for some studies referenced.

Analyte	Sampling Location/Year	Matrix	Mean Concentration	Ref.
DONA	Gendorf, Germany (2008)	Grass	0.06–0.16 ng/g (*n* = 3)	LfU [71]
	Öttinger Forest, Germany (2010)	Deer (liver)	0.6–1.5 ng/g (*n* = 6)	LfU [86]
	Tianjin, China (2017)	Locusts	0.21 ng/g (*n* = 1) ^1^	Lan et al. [87]
6:2 Cl-PFESA	Ittoqqortoormiit, Greenland (2012)	Polar bear (liver)	0.27 ng/g (*n* = 8)	Gebbink et al. [83]
	Ittoqqortoormiit, Greenland (2013)	Ringed seal (liver)	0.045 ng/g (*n* = 10)	Gebbink et al. [83]
	Tasiilaq, Greenland (2013)	Killer whale (liver)	0.023 ng/g (*n* = 5)	Gebbink et al. [83]
	Xiaoqing River, China (2014)	Crucian carp (blood)	43.0 ng/g (*n* = 30)	Shi et al. [88]
	Tangxun Lake, China (2014	Crucian carp (blood)	20.3 ng/g (*n* = 13)	Shi et al. [88]
	Huantai, China (2016)	Black-spotted frog (kidney)	7.92 ng/g (*n* = 29)	Cui et al. [89]
	Heilongjiang, China (2018)	Siberian tiger (blood)	0.08 ng/mL (*n* = 116)	Wang et al. [90]
HFPO-DA	Xiaoqing River, China (2015)	Common carp (liver)	1.37 ng/g (*n* = 15) ^2^	Pan et al. [79]
	Dordrecht, Netherlands (2016)	Deciduous leaves	4.3–86 ng/g (*n* = 5)	Brandsma et al. [34]
	Dordrecht, Netherlands (2016)	Grass	1.0–27 ng/g (*n* = 5)	Brandsma et al. [34]
	Wilmington, North Carolina (2018)	Striped bass (blood)	1.9 ng/mL (*n* = 28)	Guillette et al. [91]
HFPO-TA	Xiaoqing River, China (2015)	Common carp (liver)	587 ng/g (*n* = 15) ^2^	Pan et al. [79]
	Huantai, China (2016)	Black-spotted frog (kidney)	59.3 ng/g (*n* = 4)	Cui et al. [89]

^1^ Pooled sample consisting of > 10 individuals. ^2^ Median concentration reported.

**Table 2:** Average values for half-lives of selected PFAS in blood by country. Values for half-lives reported as geometric mean in years unless otherwise denoted. Values in parentheses represent 95% CI unless otherwise denoted.

Country	Population	PFOA	PFOS	PFHxS	PFHxA	PFHpA	PFHpS	PFPeS	PFBS	5:3 FTCA	6:2 Cl-PFESA
Sweden [122]	General population exposed to contaminated water	2.7 (2.5–2.9)	3.4 (3.1–3.7)	5.3 (4.6–6.0)							
Sweden [123]	Airport employees exposed to AFFF in drinking water	1.8 (1.4–2.3) ^1^	2.9 (1.7–9.6) ^1^	2.9 (2.1–4.5) ^1^	1.6 (NA) ^1^	62 (51–80) ^1,2^	1.5 (0.8–6.3) ^1^	0.6 (0.5–1.0) ^1^	44 (37–55) ^1,2^		
Arnsberg, Germany [128]	General population exposed to PFOA in drinking water	3.3 (1.0–14.7) ^3^									
United States [93]	3M retirees	3.5 (3.0–4.1)	4.8 (4.0–5.8)	7.3 (5.8–9.2)							
Alabama, United States [129]	Contaminated water near PFAS manufacturer	3.9 (3.5–4.1) ^3^	3.3 (3.0–3.6) ^3^	15.5 (13.4–17.6) ^3^							
United States [130]	3M employees								25.8 (16.6–40.2) ^2^		
Europe [102]	Ski wax technicians				32 (14–49) ^2,3^	70 (31–1223) ^2,3^				43 (25–96) ^2,3^	
Shandong, China [73]	Metal platers		7.7 (3.0–19.1) ^1,3^								18.5 (10.1–56.4) ^1,3^

^1^ Arithmetic mean reported. ^2^ Half-lives reported in days. ^3^ Values in parentheses represent range.

## Data Availability

No new data were created or analyzed in this study. Data sharing is not applicable to this article.

## Supplementary Materials

The following are available online at https://www.mdpi.com/1422-0067/22/3/995/s1, Table S1: Names, acronyms, and CASRN of PFAS discussed in review article.

## Abbreviations

PFAS	Per- and polyfluoroalkyl substances
PFAA	Perf luoroalkyl acid
PFCA	Perfluorocarboxylic acid
PFSA	Perfluorosulfonic acid
FTOH	Fluorotelomer alcohol
FTCA	Fluorotelomer carboxylic acid
FTS	Fluorotelomer sulfonic acid
PFECA	Perfluoroethercarboxylic acid
Cl-PFECA	Chlorinated polyfluoroethercarboxylic acid
PFESA	Perfluoroethersulfonic acid
Cl-PFESA	Chlorinated polyfluoroethersulfonic acid
PAP	Perfluoroalkyl phosphate ester
diPAP	Perfluoroalkyl phosphate diester
LC–MS/MS	Liquid chromatography-tandem mass spectrometry
GC–MS	Gas chromatography-mass spectrometry
HRMS	High resolution mass spectrometry
ESI	Electrospray ionization
LOD	Limit of detection
LOQ	Limit of quantitation
ECF	Electrochemical fluorination
AFFF	Aqueous firefighting foam
FPP	Fluorochemical production plant
WWTP	Wastewater treatment plant
PPARα	Peroxisome proliferator-activated receptor α
mPPARα	Murine peroxisome proliferator-activated receptor α
hPPARα	Human peroxisome proliferator-activated receptor α
