# Supplementary material for: Legacy and Emerging Per- and Polyfluoroalkyl Substances: Analytical Techniques, Environmental Fate, and Health Effects
Source: Int J Mol Sci. 2021 Jan 20;22(3):995. doi: 10.3390/ijms22030995 (PMC7863963; doi:10.3390/ijms22030995)
Supplement: Supplementary file 1 [file ijms-22-00995-s001.pdf]

International Journal of *Molecular Sciences*

MDPI

SUPPLEMENTARY MATERIAL

Legacy and Emerging Per- and Polyfluoroalkyl Substances: Environmental Contamination and Human Exposure

Richard A. Brase [1,2], Elizabeth J. Mullin [1] and David C. Spink [1,2,*]

[1] Laboratory of Organic Analytical Chemistry, Wadsworth Center, New York State Department of Health, Albany, NY 12237, Unites States; richard.brase@health.ny.gov (R.A.B.); elizabeth.mullin@health.ny.gov (E.J.M.); david.spink@health.ny.gov (D.C.S.)

[2] Department of Environmental Health Sciences, School of Public Health, University at Albany, State University of New York, Rensselaer, NY 12144, Unites States; rbrase@albany.edu (R.A.B.); dspink@albany.edu (D.C.S.)

* Correspondence: david.spink@health.ny.gov

Supplementary Table 1. Names, acronyms, and CASRN of PFAS discussed in review article.

**Analyte**	**Acronym**	**CASRN**
perfluorobutanoic acid	PFBA	375-22-4
perfluorohexanoic acid	PFHxA	307-24-4
perfluoroheptanoic acid	PFHpA	375-85-9
perfluorooctanoic acid	PFOA	335-67-1
perfluorononanoic acid	PFNA	375-95-1
perfluorodecanoic acid	PFDA	335-76-2
perfluoroundecanoic acid	PFUdA	2058-94-8
perfluorododecanoic acid	PFDoA	307-55-1
perfluorobutanesulfonic acid	PFBS	375-73-5
perfluoropentanesulfonic acid	PFPeS	2706-91-4
perfluorohexanesulfonic acid	PFHxS	355-46-4
perfluoroheptanesulfonic acid	PFHpS	375-92-8
perfluorooctanesulfonic acid	PFOS	1763-23-1
perfluorooctane sulfonamide	FOSA	754-91-6
N-ethyl perfluorooctane sulfonamido acetic acid	N-EtFOSAA	2991-50-6
N-methyl perfluorooctane sulfonamido acetic acid	N-MeFOSAA	2355-31-9
perfluoroethylcyclohexanesulfonic acid	PFECHS	335-24-0
hexafluoropropylene oxide dimer acid	HFPO-DA	13252-13-6
hexafluoropropylene oxide trimer acid	HFPO-TA	13252-14-7
4,8-dioxa-3H-perfluorononanoic acid	DONA	958445-448 [1]
5:3 fluorotelomer carboxylic acid	5:3 FTCA	914637-49-3
8:2 fluorotelomer sulfonic acid	8:2 FTS	39108-34-4
6:2 chlorinated polyfluoroethersulfonic acid	6:2 Cl-PFESA	73606-19-6
8:2 chlorinated polyfluoroethersulfonic acid	8:2 Cl-PFESA	83329-89-9

[1] CASRN listed for ammonium salt of DONA. Also available as sodium salt (no CASRN).
